# Effectiveness of web-based educational videos on enhancing diabetes knowledge and empowerment among type 2 diabetes patients in primary care: A randomized controlled trial

**DOI:** 10.1371/journal.pone.0355192

**Published:** 2026-08-07

**Authors:** Hui Zhu Thew, Poh Ying Lim, Tatt Quan Tan, Jun Hui Goh, Amelia Qiao Rou Yap, Ja Shen Choy, Chee Han Ng

**Affiliations:** 1 Department of Family Medicine, Faculty of Medicine and Health Sciences, Universiti Putra Malaysia, Serdang, Selangor, Malaysia; 2 Department of Community Medicine, Faculty of Medicine and Health Sciences, Universiti Putra Malaysia, Serdang, Selangor, Malaysia; 3 Faculty of Medicine and Health Sciences, Universiti Putra Malaysia, Serdang, Selangor, Malaysia; Cardiff University, UNITED KINGDOM OF GREAT BRITAIN AND NORTHERN IRELAND

## Abstract

**Objective:**

This study evaluated the effectiveness of web-based educational video module on diabetes knowledge, empowerment, and clinical outcomes among adults with poorly controlled type 2 diabetes in a primary care setting.

**Methods:**

A single-centre, parallel-group, randomised controlled trial was conducted at a public primary care clinic in Malaysia. Adults aged 18 years or older with type 2 diabetes for at least six months and poor glycaemic control were randomly assigned to receive either a web-based educational video intervention or standard care. The intervention consisted of culturally adapted, multilingual educational videos covering diabetes self-care, medication use, diet, physical activity, and complication prevention, supported by monthly follow-up reminders. Primary outcomes were diabetes knowledge and empowerment. Secondary outcomes included HbA1C, blood pressure, fasting lipid profile, body mass index, and waist circumference. Outcomes were assessed at baseline, three months, and six months.

**Results:**

A total of 232 participants were randomised into intervention and control groups (n = 116 each), with mean age of 59 years old, slight male predominance (53.0%) and mostly Malay (43.1%), with obesity (51.7%). Following the web-based intervention, participants demonstrated significant improvements in diabetes knowledge (β = 23.74, 95% CI: 20.57–26.91) and empowerment (β = 11.53, 95% CI: 9.98–13.09) compared with those receiving standard care at both three and six months (p < 0.001). Improvements in HbA1C, blood pressure, fasting lipid profile, body mass index, and waist circumference were also observed in the intervention group relative to controls (p < 0.05).

**Conclusion:**

A culturally adapted, web-based educational video module was associated with improvements in diabetes knowledge, empowerment, and cardiometabolic outcomes among adults with poorly controlled type 2 diabetes. These findings support the use of web-based education as a scalable adjunct to standard diabetes care in primary care settings.

## Introduction

Diabetes mellitus remains a major global public health challenge and is a leading contributor to morbidity and mortality worldwide. In 2019, diabetes was responsible for approximately 1.5 million deaths globally. The burden is particularly pronounced in Southeast Asia, where the International Diabetes Federation estimated that more than 34 million adults were living with diabetes, with projections rising to 55 million by 2035 [1]. In Malaysia, the National Health and Morbidity Survey (NHMS) 2023 reported an increasing trend in diabetes prevalence from 11.2% in 2011 to 15.6% in 2023. Among adults who were aware that they had diabetes, more than half (56%) were reported not to have good blood sugar control. In the NHMS 2023 technical report, raised blood glucose among those not known to have diabetes was defined as fasting blood sugar ≥7.0 mmol/L or random blood sugar ≥11.1 mmol/L during the field survey [2].

Diabetes self-management education is a cornerstone of long-term disease control. Educational interventions are commonly conceptualised within the knowledge-attitude-practice framework, whereby improved understanding of the disease influences attitudes, promotes behavioural change, and ultimately improves clinical outcomes [3,4]. Numerous studies have consistently demonstrated that structured diabetes education can improve self-care behaviours and glycaemic outcomes. However, in routine primary care practice, the delivery of comprehensive education is frequently constrained by limited consultation time, workforce shortages, and logistical barriers. These challenges were further amplified during the COVID-19 pandemic, highlighting the need for alternative models of care [5,6].

Technology-assisted and web-based educational interventions have been shown to improve diabetes-related outcomes, including modest reductions in glycosylated haemoglobin (HbA1C), while offering advantages in scalability, accessibility and cost-effectiveness [7–9]. However, evidence from Malaysian primary care settings remains limited, particularly among patients with persistently poor glycaemic control.

To address this gap, the present study evaluated a culturally adapted, multilingual web-based educational video intervention designed for adults with type 2 diabetes receiving care in Malaysian primary care clinic. The primary objective was to assess the effectiveness of the intervention on diabetes knowledge and empowerment. Secondary objectives were to evaluate changes in clinical outcomes, including HbA1C, blood pressure, fasting lipid profile, body mass index (BMI), and waist circumference, over a six-month follow-up period.

## Methods

### Study design and Setting

This single-centre, parallel-group, randomised controlled trial was conducted at Cheras Baru Health Clinic, a public primary care facility in Malaysia. The study was reported in accordance with the Consolidated Standards of Reporting Trials (CONSORT) guidelines (S1 Checklist). The full study protocol is publicly accessible as shown in S2 File [10].

### Ethical Considerations

The study was conducted in accordance with the Declaration of Helsinki and the Malaysian Good Clinical Practice Guideline. Ethical approval was obtained from the Medical Research and Ethics Committee (MREC) and registered with the National Medical Research Registry (NMRR). The trial was prospectively registered with the Thai Clinical Trials Registry on 21 March 2023, with the identifying number of TCTR20230321005 (https://www.thaiclinicaltrials.org/show/TCTR20230321005).

### Participants

**Fig 1:**
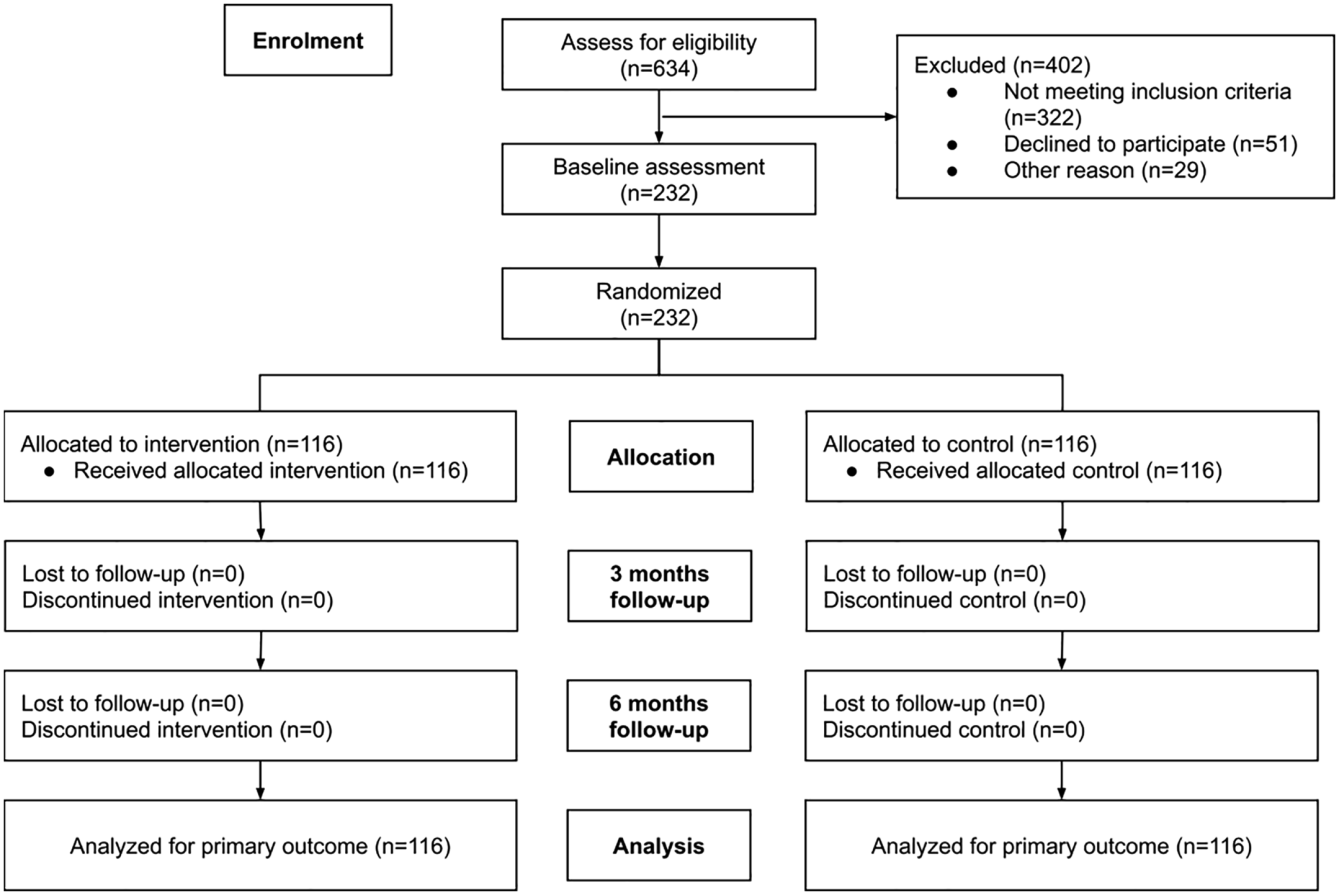
CONSORT flow diagram of study recruitment and randomisation. Following recruitment, 232 participants with poorly controlled type 2 diabetes were randomised to receive either access to a series of web-based educational videos or standard care consisting of a diabetes education session during routine clinic follow-up.

A total of 634 patients were screened for eligibility, of whom 402 were excluded. Eligible participants were adults aged 18 years or older with a diagnosis of type 2 diabetes for at least six months, poor glycaemic control, proficiency in the Malay language, and access to home internet. Only one participant per household was recruited. Poor glycaemic control was defined as HbA1c > 8.0%. This threshold was selected to identify patients with clearly suboptimal glycaemic control, as the Malaysian Clinical Practice Guidelines recommend individualised HbA1c targets, with HbA1c < 7.0% being appropriate for most adults with type 2 diabetes [11]. Patients with HbA1c > 8.0% were therefore considered an appropriate target population for an educational intervention aimed at improving diabetes self-management and clinical outcomes. Exclusion criteria included acute medical illness, psychiatric illness, dependence on nursing care, or intellectual disability. This study was conducted from 1 November 2023–31 May 2024. Ultimately, 232 participants provided written informed consent and were enrolled (Fig. 1).

### Randomisation and blinding

Participants were randomised in a 1:1 ratio to either the intervention group (n = 116) or control group (n = 116) using block randomisation. Allocation concealment was ensured through the use of sequentially numbered, sealed, opaque envelopes prepared by an independent researcher. Due to the nature of the intervention, participant blinding was not feasible; however, outcome assessors and data analysts were blinded to group allocation. To maintain outcome assessor blinding, objective clinical data were extracted directly from the laboratory and quantitative fields of the electronic medical records by independent researchers. These researchers did not have access to the consultation notes where group allocation might have been inadvertently documented by treating clinicians.

### Intervention

Participants in the intervention group received access to a series of web-based educational videos, while those in the control group received standard care, consisting of a routine diabetes education during clinic follow-up. The educational videos were developed by a multidisciplinary team and underwent validation by content experts and patients. Topics included glycaemic targets, medication use, self-monitoring of blood glucose, hypoglycaemia management, dietary practices, physical activity, eye and dental care, and complication prevention. Videos were available in Malay, English, Chinese and Tamil. Participants were encouraged to view all videos, with adherence supported through reminder phone calls every four weeks.

The educational video scripts were developed and reviewed by a multidisciplinary team comprising two family medicine specialists, one ophthalmologist, one pharmacist, two endocrinologists, one public health specialist, one rehabilitation specialist, one dietician, and three patient representatives from the Malay, Chinese, and Indian ethnic groups. The patient representatives involved in the review and validation process were independent from the trial participants. Following completion of the scripts, all experts and patient representatives reviewed the 12 video topics and completed a structured content validation questionnaire. Each topic was assessed for relevance and clarity using a three-point scale modified from a published content validity tool, where 1 indicated ‘not relevant’ or ‘not clear,’ 2 indicated ‘somewhat relevant’ or ‘somewhat clear,’ and 3 indicated ‘highly relevant’ or ‘highly clear.’ The content validity index of the video content was 0.80, indicating acceptable content validity. The English version was subsequently translated into Malay, Chinese, and Tamil by the research team. A pilot study was conducted among five respondents for each language version. Minor grammatical errors and difficult terminology were identified and revised before finalisation. Reliability testing was then conducted for the Malay, Chinese, and Tamil versions. All language versions demonstrated satisfactory internal consistency, with Cronbach’s alpha coefficients exceeding the commonly accepted threshold of 0.70 [10].

### Data collection and outcome measures

At baseline, participants completed a self-administered questionnaire in the Malay language, comprising three sections: (1) sociodemographic characteristics (age, gender, ethnicity, education level, marital status, employment status, income category, smoking status, obesity status and history of hospitalization due to complications); (2) the Diabetes Knowledge Test (DKT), a validated instrument assessing diabetes knowledge; (3) the Diabetes Empowerment Scale (DES-28), a validated tool measuring psychosocial self-efficacy related to diabetes empowerment. The Malay versions of DKT and DES-28 demonstrated Cronbach’s alpha of 0.573 and 0.920 respectively [12,13]. The DKT score was calculated as the percentage of correct responses, with possible scores ranging from 0 to 100. Higher scores indicate better diabetes knowledge. For the DES-28, it consists of 28 items across three subscales. Each item is rated using a 5-point Likert scale, where 1 indicates “strongly disagree” and 5 indicates “strongly agree”. The total score ranges from 28 to 140, with higher scores indicating greater diabetes-related empowerment. Malay was used as the medium of administration as it is the official language in Malaysia.

Clinical outcomes, including HbA1c level, blood pressure reading, fasting lipid profile which consisted of low-density lipoprotein (LDL), high-density lipoprotein (HDL) and triglyceride level, BMI and waist circumference were extracted from participants’ medical records. Follow-up assessments were conducted at three months and six months using the same questionnaires and clinical data extraction.

### Sample Size

The sample size was calculated using the two-sample means comparison formula. A previous study reported the knowledge score differences between intervention and control groups were 0.7 ± 2.21 and −0.3 ± 2.33 respectively [9]. Assuming a two-sided alpha level of 0.05 with a power of 80% and 30% attrition, a total of 232 participants (116 per group) was required.

### Statistical analysis

Data analysis was performed using SPSS version 29.0 according to the intention-to-treat (ITT) principle. Between-group comparisons were conducted using independent t-tests, while within-group changes were assessed using paired t-tests. Longitudinal intervention effects were analyzed using generalized estimating equations (GEE) with adjustment for baseline variables that differed significantly between groups. GEE was selected because outcomes were measured repeatedly in the same participants at baseline, three months, and six months, and this approach accounts for within-participant correlation over time while providing population-averaged estimates of intervention effects. Different types of working correlation structures (independence, exchangeable, and autoregressive) were examined as part of model diagnostics. The final model was selected based on the lowest Quasi-likelihood under the Independence model Criterion (QIC), ensuring the best balance between model fit and parsimony. Robust standard errors were used to reduce the impact of possible misspecification of the working correlation structure. *P*-value < 0.05 was considered statistically significant.

## Results

Throughout the study period, a total of 232 data was collected at baseline. As there was zero attrition at both three-month and six-month follow-up assessments, all datasets for the 232 participants were complete.

### SBaseline characteristics

A total of 232 participants were randomised, with 116 allocated to each group. Baseline characteristics are summarised in Table 1. Significant differences between groups were observed for age, educational level, smoking status, obesity prevalence, and history of diabetes-related hospitalisation (p < 0.05). The intervention group was older (61.2 ± 10.5 vs 57.6 ± 11.0 years) and had a slightly higher baseline diabetes empowerment score (95.3 ± 5.2 vs 93.8 ± 5.0). No significant differences were observed for baseline diabetes knowledge or clinical outcomes (Table 2).

**Table 1:** Baseline Comparison of Sociodemographic Characteristics of Participants by Groups.

Sociodemographic Characteristics	Control Group (n = 116)	Intervention Group (n = 116)	Total (n = 232)	SMD	*p*-value
**Age, years**	57.57 ± 11.016	61.24 ± 10.523	59.41 ± 10.905	0.34	0.010^*^
**Gender, n (%)**				0.05	0.693
**Male**	60 (51.7)	63 (54.3)	123 (53.0)		
**Female**	56 (48.3)	53 (45.7)	109 (47.0)		
**Ethnicity, n (%)**				0.48	0.056
**Malay**	42 (36.2)	58 (50.0)	100 (43.1)		
**Chinese**	55 (47.4)	35 (30.2)	90 (38.8)		
**Indian**	18 (15.5)	21 (18.1)	39 (16.8)		
**Others**	1 (0.9)	2 (1.7)	3 (1.3)		
**Education Level, n (%)**				0.41	0.021^*^
**No formal education/ Primary education**	36 (31.0)	27 (23.3)	63 (27.2)		
**Secondary education**	67 (57.8)	60 (51.7)	127 (54.7)		
**Tertiary education**	13 (11.2)	29 (25.0)	42 (18.1)		
**Marital Status, n (%)**				0.21	0.138
**Single/ Widow/ Widower**	9 (7.8)	16 (13.8)	25 (10.8)		
**Married**	107 (92.2%)	100 (86.2)	207 (89.2)		
**Employment Status, n (%)**				0.12	0.348
**No**	73 (62.9)	66 (56.9)	139 (59.9)		
**Yes**	43 (37.1)	50 (43.1)	93 (40.1)		
**Income Category, n (%)**				0.24	0.119
**B40 (RM0 - RM4849)**	115 (99.1)	110 (94.8)	225 (97.0)		
**M40 (RM4850 - RM10,959)**	1 (0.9)	6 (5.2)	7 (3.0)		
**Smoking Status, n (%)**				0.34	0.012^*^
**No**	87 (75.0)	69 (59.5)	156 (67.2)		
**Yes/ Used to smoke but quit less than six months ago/ Have smoked and quit for more than six months**	29 (25.0)	47 (40.5)	76 (32.8)		
**Obesity Status, n (%)**				0.52	0.015^*^
**No**	31 (26.7)	47 (40.5)	78 (33.6)		
**Yes**	71 (61.2)	49 (42.2)	120 (51.7)		
**Not sure**	14 (12.1)	20 (17.2)	34 (14.7)		
**History of Hospitalization due to Complications, n (%)**				0.34	0.012^*^
**No**	86 (74.1)	68 (58.6)	154 (66.4)		
**Yes**	30 (25.9)	48 (41.4)	78 (33.6)		

Data are mean ± SD or n (%), SMD: Standardised Mean Differences

* *p* < 0.05

**Table 2:** Baseline Comparison of Diabetes Knowledge, Empowerment and Clinical Outcomes of Participants by Groups.

Outcomes	Control Group (n = 116)	Intervention Group (n = 116)	Total (n = 232)	SMD	*p*-value
**Diabetes Knowledge, %**	52.62 ± 11.764	53.39 ± 11.917	53.01 ± 11.822	0.07	0.623
**Diabetes Empowerment**	93.78 ± 4.982	95.33 ± 5.190	94.55 ± 5.135	0.31	0.021^*^
**Clinical Outcomes**					
**HbA1c, %**	8.30 (1.10)	8.55 (1.20)	8.50 (1.10)	0.29	0.629
**Blood Pressure, mmHg**					
**Systolic**	137.34 ± 14.35	135.22 ± 12.06	136.28 ± 13.27	0.16	0.224
**Diastolic**	78.68 ± 8.36	77.94 ± 8.53	78.58 ± 9.44	0.09	0.873
**Fasting Lipid Profile**					
**LDL, mmol/L**	2.20 (0.90)	2.45 (1.20)	2.30 (1.10)	0.31	0.143
**HDL, mmol/L**	1.35 ± 0.314	1.37 ± 0.280	1.35 ± 0.297	0.07	0.924
**Triglyceride, mmol/L**	1.70 (0.70)	1.76 (0.70)	1.70 (0.70)	0.12	0.826
**BMI, kg/m**^**2**^	25.95 (5.45)	25.30 (5.90)	25.50 (5.77)	0.15	0.261
**Waist Circumference, cm**	94.32 ± 13.666	91.32 ± 14.459	92.82 ± 14.118	0.21	0.106

Data are mean ± SD or median (IQR), SMD: Standardised Mean Differences

* *p* < 0.05

### Intervention effects

As shown in Table 3, the mean diabetes knowledge and empowerment scores have substantial improvement in the intervention group as compared to the control group throughout the six-month study period.

**Table 3:** Comparison of Mean Diabetes Knowledge and Empowerment Scores by Groups Across All Time Points.

Outcomes	Time Points	Control Group (n = 116)	Intervention Group (n = 116)
**Mean of Diabetes Knowledge Score, %**	Baseline	52.62	53.39
	3 months	54.35	79.03
	6 months	57.63	82.13
**Mean of Diabetes Empowerment Score**	Baseline	93.78	95.33
	3 months	94.15	104.70
	6 months	95.22	108.30

The effectiveness of the intervention module on diabetes knowledge, empowerment, and clinical outcomes is presented in Table 4. Detailed final models adjusted for significant baseline covariates (age, educational level, smoking status, obesity status, and history of hospitalisation) are provided in S3 Table through S12 Table. At three and six months, participants in the intervention group demonstrated greater improvements in diabetes knowledge and empowerment compared with those receiving standard care (p < 0.001). Regarding glycaemic control, HbA1C levels declined significantly in the intervention group relative to controls, with a mean reduction of 0.89% at six months (p < 0.001). Furthermore, significant improvements were observed in systolic and diastolic blood pressure, BMI and waist circumference in the intervention group at both follow-up time points (p < 0.05). Favourable changes in lipid parameters were also noted, including reductions in LDL cholesterol and triglycerides and increases in HDL cholesterol, in the intervention group at three and six months (p < 0.001).

**Table 4:** Effectiveness of Intervention Module on Diabetes Knowledge, Empowerment and Clinical Outcomes at 3 and 6 months.

Outcomes	Intervention x 3 months				Intervention x 6 months			
	β	95% CI		*p*-value	β	95% CI		*p*-value
**Diabetes Knowledge**	23.912	20.737	27.088	<0.001^***^	23.736	20.566	26.907	<0.001^***^
**Diabetes Empowerment**	9.000	7.853	10.147	<0.001^***^	11.534	9.980	13.089	<0.001^***^
**HbA1C**	−0.536	−0.640	−0.432	<0.001^***^	−0.887	−1.028	−0.746	<0.001^***^
**Blood Pressure**								
**Systolic**	−2.828	−4.123	−1.532	<0.001^***^	−4.379	−5.971	−2.788	<0.001^***^
**Diastolic**	−2.060	−3.536	−0.584	0.006^**^	−2.207	−4.146	−0.268	0.026^*^
**Fasting Lipid Profile**								
**LDL**	−0.300	−0.364	−0.236	<0.001^***^	−0.474	−0.561	−0.388	<0.001^***^
**HDL**	0.093	0.052	0.134	<0.001^***^	0.150	0.096	0.204	<0.001^***^
**Triglyceride**	−0.203	−0.264	−0.143	<0.001^***^	−0.297	−0.378	−0.215	<0.001^***^
**BMI**	−0.554	−0.748	−0.360	<0.001^***^	−1.077	−1.361	−0.793	<0.001^***^
**Waist Circulference**	−1.345	−1.798	−0.892	<0.001^***^	−2.767	−3.485	−2.049	<0.001^***^

* *p* < 0.05, ** *p* < 0.01, *** *p* < 0.001

QIC and QICC values: Model 1 (knowledge): 92473, 92473; Model 2 (empowerment); 11875, 11635; Model 3 (HbA1C): −419264393731, 720; Model 4 (systolic blood pressure): 97906, 97906; Model 5 (diastolic blood pressure): 41963, 41949; Model 6 (LDL): 591, 501; Model 7 (HDL): 111, 84; Model 8 (triglyceride): 265, 240; Model 9 (body mass index): 7760, 7732; Model 10 (waist circumference): 80952, 80919

β: Unstandardised coefficient; CI: Confidence interval; Robust estimator for covariate matrix, unstructured working correlation matrix

## Discussion

This randomised controlled trial evaluated the impact of a culturally adapted, web-based educational video intervention on diabetes-related knowledge, empowerment, and clinical outcomes among adults with poorly controlled type 2 diabetes in a Malaysian primary care setting. The findings indicate that participants receiving the intervention experienced improvements across both patient-reported and objective clinical measures over a six-month period.

A key finding of this study was the concurrent improvement in diabetes knowledge, empowerment and clinical outcomes observed in the intervention group. We consider this improvement clinically plausible despite the absence of face-to-face teaching because the educational videos were accessible to participants throughout the study period. Participants were able to review the videos repeatedly at their own pace during the 3-month and 6-month follow-up periods. In addition, the videos were structured, multilingual, culturally adapted, and developed with input from a multidisciplinary expert panel and patient representatives. While baseline diabetes knowledge and empowerment levels were comparable between groups, participants receiving the web-based education demonstrated significantly greater improvement, reflecting enhanced confidence and self-efficacy in managing their condition. This suggests that the intervention may have facilitated not only information acquisition but also meaningful behavioural engagement. The accompanying reductions in BMI and waist circumference further support the translation of educational content into lifestyle modification, consistent with the knowledge-attitude-practice framework underpinning the intervention design [14,15].

The observed reduction in HbA1C of 0.89% at six months is clinically meaningful as even modest improvements in glycaemic control are associated with reduced risks of microvascular and macrovascular complications. The magnitude of HbA1C reduction observed in this study is a clinically meaningful improvement, underscoring the potential value of digital education as a robust non-pharmacological adjunct to standard diabetes management [16,17]. In addition to glycaemic control, improvements in blood pressure and lipid profiles suggest a broader cardiometabolic benefit, which is particularly relevant given the elevated cardiovascular risk among individuals with type 2 diabetes [18].

Despite baseline imbalances between groups, whereby the intervention group was older and had a higher prevalence of prior diabetes-related hospitalisation, the intervention group remained effective after adjustment for these variables using GEE analysis. This finding suggests that language-appropriate digital education can be accessible and beneficial even among older adults and individuals with more advanced disease [19].

The multilingual and culturally tailored design of the educational videos likely contributed to participant engagement by reducing language barriers and enhancing contextual relevance within Malaysia’s multiethnic population. The short, modular video format may have further facilitated repeated viewing and flexible learning, aligning with the practical constraints faced by patients in routine primary care settings [20].

Several strengths of this study merit consideration. These include the randomised controlled design, the application of intention-to-treat analysis, and the use of expert-validated, multilingual educational content. However, several limitations should also be acknowledged. First, as a single-centre study conducted in an urban public primary care clinic, the findings may not be fully generalisable to other healthcare settings, such as the private sector, or to rural areas with poor internet connectivity. Second, the use of regular reminder phone calls to support adherence may have introduced performance bias, as this intensive level of follow-up support exceeds typical web-based interventions and may not be feasible in routine real-world clinical practice. Third, there was no objective measure of video viewing and the assessment of intervention adherence relied entirely on self-reporting, which introduces the possibility of response bias. Fourth, the reliance on self-administered questionnaires means the reported improvements, particularly in diabetes empowerment, are susceptible to social desirability bias. Finally, the Malay version of the Diabetes Knowledge Test (DKT) demonstrated low internal consistency (Cronbach’s alpha = 0.573). This suboptimal reliability may introduce measurement error, warranting a cautious interpretation of the results. Despite randomisation, several baseline characteristics differed between groups. This may reflect chance imbalance in a small-to-moderate randomised trial, particularly because block randomisation was conducted without stratification by key prognostic variables such as age, education level, smoking status, obesity status, or history of diabetes-related hospitalisation. Adjusted GEE models were used to account for these imbalances; however, statistical adjustment does not fully replace true baseline balance. Future studies may consider stratified randomisation or minimisation procedures to improve balance across important baseline characteristics.

## Conclusion

This study demonstrates that a culturally adapted, web-based educational video intervention was associated with improvements in diabetes knowledge, empowerment, and cardiometabolic outcomes among adults with poorly controlled type 2 diabetes in a primary care setting. These findings support the role of web-based education as a scalable adjunct to standard diabetes care. Future studies should explore long-term sustainability, cost-effectiveness, and scalability across diverse healthcare contexts.

## Supporting information

S1 Checklist CONSORT Checklist. (PDF)

S2 File Study Protocol. (PDF)

S3 Table Final Model of Diabetes Knowledge. (DOCX)

S4 Table Final Model of Diabetes Empowerment. (DOCX)

S5 Table Final Model of HbA1C. (DOCX)

S6 Table Final Model of Systolic Blood Pressure. (DOCX)

S7 Table Final Model of Diastolic Blood Pressure. (DOCX)

S8 Table Final Model of LDL. (DOCX)

S9 Table Final Model of HDL. (DOCX)

S10 Table Final Model of Triglyceride. (PDF)

S11 Table Final Model of BMI. (PDF)

S12 Table Final Model of Waist Circumference. (PDF)
