# Supplementary material for: Effectiveness of web-based educational videos on enhancing diabetes knowledge and empowerment among type 2 diabetes patients in primary care: A randomized controlled trial
Source: PLoS One. 2026 Aug 7;21(8):e0355192. doi: 10.1371/journal.pone.0355192 (PMC13450740; doi:10.1371/journal.pone.0355192)
Supplement: Supplementary file 6 [file pone.0355192.s003.docx]

**S3 Table**. **Final Model of Diabetes Knowledge.**

| **Parameter** | **β** | **SE** | **95% CI** | ***p*-value** |
| --- | --- | --- | --- | --- |
| **Intercept** | 59.468 | 9.752 | 40.355, 78.582 | <0.001^***^ |
| **Intervention Group** | -0.238 | 1.586 | -3.346, 2.870 | 0.881 |
| **Control Group** | Ref |  |  |  |
| **6 months** | 5.006 | 1.056 | 2.878, 7.134 | <0.001^***^ |
| **3 months** | 1.728 | 0.935 | -0.104, 3.560 | 0.064 |
| **Baseline** | Ref |  |  |  |
| **Intervention Group at 6 months** | 23.736 | 1.618 | 20.566, 26.907 | <0.001^***^ |
| **Intervention Group at 3 months** | 23.912 | 1.620 | 20.737, 27.088 | <0.001^***^ |
| **Intervention Group at Baseline** | Ref |  |  |  |
| **Control Group at 6 months** | Ref |  |  |  |
| **Control Group at 3 months** | Ref |  |  |  |
| **Control Group at Baseline** | Ref |  |  |  |

^***^*p*<0.001

β: Unstandardized coefficient; SE: Standard error; CI: Confidence interval; Ref: Reference
