# Supplementary material for: Effectiveness of web-based educational videos on enhancing diabetes knowledge and empowerment among type 2 diabetes patients in primary care: A randomized controlled trial
Source: PLoS One. 2026 Aug 7;21(8):e0355192. doi: 10.1371/journal.pone.0355192 (PMC13450740; doi:10.1371/journal.pone.0355192)
Supplement: Supplementary file 7 [file pone.0355192.s004.docx]

**S4 Table. Final Model of Diabetes Empowerment.**

| **Parameter** | **β** | **SE** | **95% CI** | ***p*-value** |
| --- | --- | --- | --- | --- |
| **Intercept** | 32.786 | 13.381 | 6.560, 59.013 | 0.014^*^ |
| **Intervention Group** | 0.314 | 0.664 | -0.986, 1.615 | 0.636 |
| **Control Group** | Ref |  |  |  |
| **6 months** | 1.440 | 0.442 | 0.574, 2.306 | 0.001^***^ |
| **3 months** | 0.371 | 0.336 | -0.288, 1.029 | 0.270 |
| **Baseline** | Ref |  |  |  |
| **Intervention Group at 6 months** | 11.534 | 0.793 | 9.980, 13.089 | <0.001^***^ |
| **Intervention Group at 3 months** | 9.000 | 0.586 | 7.853, 10.147 | <0.001^***^ |
| **Intervention Group at Baseline** | Ref |  |  |  |
| **Control Group at 6 months** | Ref |  |  |  |
| **Control Group at 3 months** | Ref |  |  |  |
| **Control Group at Baseline** | Ref |  |  |  |

^*^*p*<0.05; ^***^*p*<0.001

β: Unstandardized coefficient; SE: Standard error; CI: Confidence interval; Ref: Reference
