# Supplementary material for: Effectiveness of web-based educational videos on enhancing diabetes knowledge and empowerment among type 2 diabetes patients in primary care: A randomized controlled trial
Source: PLoS One. 2026 Aug 7;21(8):e0355192. doi: 10.1371/journal.pone.0355192 (PMC13450740; doi:10.1371/journal.pone.0355192)
Supplement: Supplementary file 8 [file pone.0355192.s005.docx]

**S5 Table. Final Model of Glycosylated Haemoglobin (HbA1c).**

| **Parameter** | **β** | **SE** | **95% CI** | ***p*-value** |
| --- | --- | --- | --- | --- |
| **Intercept** | 9.113 | - | - | <0.001^***^ |
| **Intervention Group** | 0.088 | 2.453 | -4.720, 4.897 | 0.971 |
| **Control Group** | Ref |  |  |  |
| **6 months** | 0.229 | - | - | <0.001^***^ |
| **3 months** | 0.230 | - | - | <0.001^***^ |
| **Baseline** | Ref |  |  |  |
| **Intervention Group at 6 months** | -0.887 | 0.072 | -1.028, -0.746 | <0.001^***^ |
| **Intervention Group at 3 months** | -0.536 | 0.053 | -0.640, -0.432 | <0.001^***^ |
| **Intervention Group at Baseline** | Ref |  |  |  |
| **Control Group at 6 months** | Ref |  |  |  |
| **Control Group at 3 months** | Ref |  |  |  |
| **Control Group at Baseline** | Ref |  |  |  |

^***^*p*<0.001

β: Unstandardized coefficient; SE: Standard error; CI: Confidence interval; Ref: Reference
