# Supplementary material for: Effectiveness of web-based educational videos on enhancing diabetes knowledge and empowerment among type 2 diabetes patients in primary care: A randomized controlled trial
Source: PLoS One. 2026 Aug 7;21(8):e0355192. doi: 10.1371/journal.pone.0355192 (PMC13450740; doi:10.1371/journal.pone.0355192)
Supplement: Supplementary file 9 [file pone.0355192.s006.docx]

**S6 Table. Final Model of Systolic Blood Pressure.**

| **Parameter** | **β** | **SE** | **95% CI** | ***p*-value** |
| --- | --- | --- | --- | --- |
| **Intercept** | 137.607 | 17.540 | 103.229, 171.984 | <0.001^***^ |
| **Intervention Group** | -0.697 | 1.928 | -4.476, 3.081 | 0.718 |
| **Control Group** | Ref |  |  |  |
| **6 months** | -0.026 | 0.548 | -1.100, 1.048 | 0.962 |
| **3 months** | 0.086 | 0.468 | -0.831, 1.004 | 0.854 |
| **Baseline** | Ref |  |  |  |
| **Intervention Group at 6 months** | -4.379 | 0.812 | -5.971, -2.788 | <0.001^***^ |
| **Intervention Group at 3 months** | -2.828 | 0.662 | -4.123, -1.532 | <0.001^***^ |
| **Intervention Group at Baseline** | Ref |  |  |  |
| **Control Group at 6 months** | Ref |  |  |  |
| **Control Group at 3 months** | Ref |  |  |  |
| **Control Group at Baseline** | Ref |  |  |  |

^***^*p*<0.001

β: Unstandardized coefficient; SE: Standard error; CI: Confidence interval; Ref: Reference
