# Supplementary material for: Effectiveness of web-based educational videos on enhancing diabetes knowledge and empowerment among type 2 diabetes patients in primary care: A randomized controlled trial
Source: PLoS One. 2026 Aug 7;21(8):e0355192. doi: 10.1371/journal.pone.0355192 (PMC13450740; doi:10.1371/journal.pone.0355192)
Supplement: Supplementary file 10 [file pone.0355192.s007.docx]

**S7 Table. Final Model of Diastolic Blood Pressure.**

| **Parameter** | **β** | **SE** | **95% CI** | ***p*-value** |
| --- | --- | --- | --- | --- |
| **Intercept** | 105.062 | 8.178 | 89.034, 121.090 | <0.001^***^ |
| **Intervention Group** | 0.391 | 1.207 | -1.973, 2.756 | 0.746 |
| **Control Group** | Ref |  |  |  |
| **6 months** | 1.767 | 0.583 | 0.625, 2.910 | 0.002^**^ |
| **3 months** | 1.810 | 0.576 | 0.682, 2.938 | 0.002^**^ |
| **Baseline** | Ref |  |  |  |
| **Intervention Group at 6 months** | -2.207 | 0.989 | -4.146, -0.268 | 0.026^*^ |
| **Intervention Group at 3 months** | -2.060 | 0.753 | -3.536, -0.584 | 0.006^**^ |
| **Intervention Group at Baseline** | Ref |  |  |  |
| **Control Group at 6 months** | Ref |  |  |  |
| **Control Group at 3 months** | Ref |  |  |  |
| **Control Group at Baseline** | Ref |  |  |  |

^*^*p*<0.05; ^**^*p*<0.01; ^***^*p*<0.001

β: Unstandardized coefficient; SE: Standard error; CI: Confidence interval; Ref: Reference
