# Supplementary material for: Effectiveness of web-based educational videos on enhancing diabetes knowledge and empowerment among type 2 diabetes patients in primary care: A randomized controlled trial
Source: PLoS One. 2026 Aug 7;21(8):e0355192. doi: 10.1371/journal.pone.0355192 (PMC13450740; doi:10.1371/journal.pone.0355192)
Supplement: Supplementary file 11 [file pone.0355192.s008.docx]

**S8 Table. Final Model of Low-Density Lipoprotein (LDL).**

| **Parameter** | **β** | **SE** | **95% CI** | ***p*-value** |
| --- | --- | --- | --- | --- |
| **Intercept** | 2.635 | 1.383 | -0.076, 5.346 | 0.057 |
| **Intervention Group** | 0.038 | 0.147 | -0.250, 0.327 | 0.794 |
| **Control Group** | Ref |  |  |  |
| **6 months** | 0.070 | 0.029 | 0.013, 0.126 | 0.016^*^ |
| **3 months** | 0.086 | 0.025 | 0.038, 0.134 | <0.001^***^ |
| **Baseline** | Ref |  |  |  |
| **Intervention Group at 6 months** | -0.474 | 0.044 | -0.561, -0.388 | <0.001^***^ |
| **Intervention Group at 3 months** | -0.300 | 0.033 | -0.364, -0.236 | <0.001^***^ |
| **Intervention Group at Baseline** | Ref |  |  |  |
| **Control Group at 6 months** | Ref |  |  |  |
| **Control Group at 3 months** | Ref |  |  |  |
| **Control Group at Baseline** | Ref |  |  |  |

^*^*p*<0.05; ^***^*p*<0.001

β: Unstandardized coefficient; SE: Standard error; CI: Confidence interval; Ref: Reference
