# Supplementary material for: Effectiveness of web-based educational videos on enhancing diabetes knowledge and empowerment among type 2 diabetes patients in primary care: A randomized controlled trial
Source: PLoS One. 2026 Aug 7;21(8):e0355192. doi: 10.1371/journal.pone.0355192 (PMC13450740; doi:10.1371/journal.pone.0355192)
Supplement: Supplementary file 12 [file pone.0355192.s009.docx]

**S9 Table. Final Model of High-Density Lipoprotein (HDL).**

| **Parameter** | **β** | **SE** | **95% CI** | ***p*-value** |
| --- | --- | --- | --- | --- |
| **Intercept** | 1.032 | 0.343 | 0.360, 1.705 | 0.003^**^ |
| **Intervention Group** | 0.000 | 0.047 | -0.093, 0.092 | 0.992 |
| **Control Group** | Ref |  |  |  |
| **6 months** | 0.010 | 0.015 | -0.020, 0.041 | 0.502 |
| **3 months** | -0.008 | 0.013 | -0.033, 0.018 | 0.550 |
| **Baseline** | Ref |  |  |  |
| **Intervention Group at 6 months** | 0.150 | 0.027 | 0.096, 0.204 | <0.001^***^ |
| **Intervention Group at 3 months** | 0.093 | 0.021 | 0.052, 0.134 | <0.001^***^ |
| **Intervention Group at Baseline** | Ref |  |  |  |
| **Control Group at 6 months** | Ref |  |  |  |
| **Control Group at 3 months** | Ref |  |  |  |
| **Control Group at Baseline** | Ref |  |  |  |

^**^*p*<0.01; ^***^*p*<0.001

β: Unstandardized coefficient; SE: Standard error; CI: Confidence interval; Ref: Reference
